# FGFR Inhibitors in Cholangiocarcinoma—A Novel Yet Primary Approach: Where Do We Stand Now and Where to Head Next in Targeting This Axis?

**DOI:** 10.3390/cells11233929

**Published:** 2022-12-05

**Authors:** Paulina Chmiel, Katarzyna Gęca, Karol Rawicz-Pruszyński, Wojciech P. Polkowski, Magdalena Skórzewska

**Affiliations:** Department of Surgical Oncology, Medical University of Lublin, 20-080 Lublin, Poland

**Keywords:** cholangiocarcinoma, CCA, FGFR, targeted therapy, therapy resistance, TKIs

## Abstract

Cholangiocarcinomas (CCAs) are rare but aggressive tumours with poor diagnosis and limited treatment options. Molecular targeted therapies became a promising proposal for patients after progression under first-line chemical treatment. In light of an escalating prevalence of CCA, it is crucial to fully comprehend its pathophysiology, aetiology, and possible targets in therapy. Such knowledge would play a pivotal role in searching for new therapeutic approaches concerning diseases’ symptoms and their underlying causes. Growing evidence showed that fibroblast growth factor/fibroblast growth factor receptor (FGF/FGFR) pathway dysregulation is involved in a variety of processes during embryonic development and homeostasis as well as tumorigenesis. CCA is known for its close correlation with the FGF/FGFR pathway and targeting this axis has been proposed in treatment guidelines. Bearing in mind the significance of molecular targeted therapies in different neoplasms, it seems most reasonable to move towards intensive research and testing on these in the case of CCA. However, there is still a need for more data covering this topic. Although positive results of many pre-clinical and clinical studies are discussed in this review, many difficulties lie ahead. Furthermore, this review presents up-to-date literature regarding the outcomes of the latest clinical data and discussion over future directions of FGFR-directed therapies in patients with CCA.

## 1. Introduction

The last decade has seen intensive developments in cancer genome research, which have become the basis for the use of specific small molecules targeting disturbed cellular processes. Dysregulation of FGFR signalling is observed in a subset of many cancers, making activated FGFRs a highly promising potential therapeutic target, supported by multiple pre-clinical studies and clinical trials [1]. The FGF/FGFR signalling pathway is mainly affected by gene amplification, gain-of-function coding mutations, and gene fusion [2]. Consequently, novel treatment opportunities have arisen, including the use of FGFR inhibitors in tumours with poor prognosis and limited treatment options, such as cholangiocarcinoma (CCA) [3,4]. Because of its rare occurrence, CCA remains a diagnostic and therapeutic challenge. CCA patients’ estimated 5-year survival rate oscillates around 5% [5]. Multiple studies proved the importance of *FGFR* gene mutations in the development of this cancer, especially *FGFR2* fusion with the essential p.V565F gate-keeper mutation [6]. Management of CCA currently is based on a surgical approach and chemotherapy which have limited effectiveness; therefore, the need for improvement is seen among clinicians and researchers. Food and drug administration (FDA) approved three main molecules for managing previously treated, unresectable, locally advanced, or metastatic, CCA with an FGFR2 fusion or another rearrangement [7,8]. However, at this phase of research, a constant interplay between cancer resistance mechanisms and novel therapies is crucial for efficient treatment [9].

This review comprehensively summarises today’s CCA management means and their limitations. Pivotal issues such as resistance, side effects, and combined therapies have been discussed with emphasis on the need for further in-depth research to increase the effectiveness of the FGFR inhibitor usage.

## 2. Cholangiocarcinoma—In a Summary

**Figure 1:**
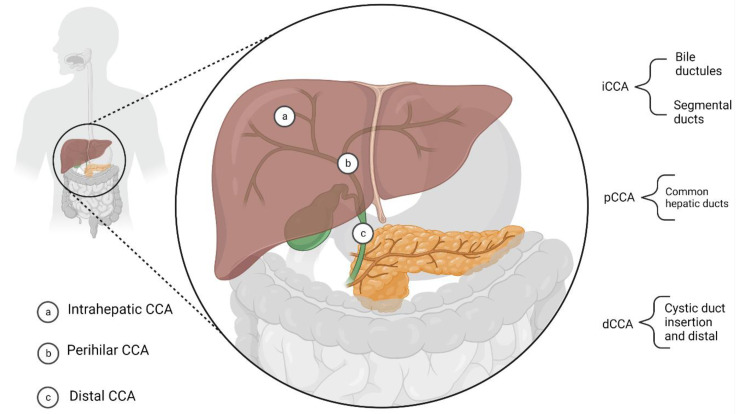
Anatomical division of cholangiocarcinoma (CCA).

CCA is a heterogeneous group of malignancies, currently being one of the most urgent issues of gastrointestinal oncology. It consists of various malignant tumours that arise from any point along the biliary ducts [3,10]. Based on the most common anatomical features, cholangiocarcinoma can be divided into intrahepatic (iCCA), perihilar (pCCA), and distal (dCCA) [11,12,13]. According to this division, iCCA arises proximally to the second-order bile ducts [14], pCCA is localized between the second-order bile ducts and the insertion of the cystic duct into the common bile duct, whereas dCCA is found distal from the cystic duct insertion [14]. Notably, pCCA and dCCA can be referred to as extrahepatic (eCCA), which corresponds to different pathogenesis and genetics from iCCA [13,15] (Figure 1).

Although CCA is the most common cancer in the biliary duct, the general incidence is relatively low and varies depending on the geographic region. CCA makes up 3% of gastrointestinal malignancies [4,16]. Based on the anatomical division, pCCA is the most common type of CCA and accounts for up to 50–60% of all CCA [4,17]. dCCA (20–30%) and iCCA (10%) occur less commonly, comprising around 9% of all primary liver cancers [18]. Furthermore, in the last few years, an increase in the incidence of iCCA and a decrease in that of eCCA has been observed [19,20]. The latest epidemiology findings showed that the incidence of iCCA increased from 0.44 to 1.18 cases per 100,000 [21]. The occurrence is the highest in the 6–7th decade of life [19]. Based on work by Banales et al., worldwide trends state that the highest incidence rate is reported in Thailand, South Korea, and China, reaching nearly 10 cases per 100,000 [3]. Lower incidence rates are reported in Western countries; however, the diagnosis there is made on more advanced stages [12]. Despite the low incidence rates of CCA, it is one of the most deadly cancers, with a median overall survival (OS) of around 20 months [22]. Late diagnosis, suboptimal treatment, and frequent tumour recurrence after resection account for only 7 to 20% of 5-year survival rates in patients with CCA [23,24,25].

The most crucial risk factor for both iCCA and eCCA is chronic inflammation and irritation of the biliary epithelium [26]. The most common and best-documented risk factors include, but are not limited to: biliary diseases (such as primary sclerosing cholangitisand primary or secondary biliary cirrhosis); cholelithiasis; cholecystitis; liver flukes; cirrhosis; alcoholic liver disease; type II diabetes; and chronic pancreatitis [27,28,29]. Fundamental differences between iCCA and eCCA are also visible in the risk factors. Often correlated with iCCA, but not with eCCA, are hepatitis B and C; tobacco use; human immunodeficiency virus (HIV); inflammatory bowel disease; obesity; and chemical toxins (dioxins, vinyl chloride, nitrosamines) [30,31]. However, despite well-defined risk factors, 50% of cases are still diagnosed without any identifiable cause [32].

CCA is often diagnosed in advanced stages, mainly due to its asymptomatic course. The most typical symptoms are painless jaundice, abdominal pain, nausea, and weight loss [33]. They are associated with an advanced stage and are particularly observed in eCCA [34]. Around 20% of iCCA are incidental findings in control ultrasonography (USG) [3]. The basis of diagnosis and perioperative management is computed tomography (CT) imaging, providing a comprehensive evaluation of the primary tumour, the relationship with adjacent structures, and the spread to other organs [35]. Other techniques used during diagnosis are magnetic resonance imaging (MRI) with cholangiopancreatography (MRCP) option, USG, or contrast-enhanced ultrasonography (CEUS) [36,37]. However, histopathological examination is required for a definite diagnosis, allowing classification and defining specific genetic aberrations [12,38].

Management of CCA strictly depends on the clinical stage, tumour features, and localization, yet eCCA and iCCA have many distinct features, especially in management and treatment. Only 25% of patients are diagnosed in the early stages with the possibility of radical surgical resection, even if the recurrence rate remains high [39]. For patients with metastatic or locally advanced disease, treatment options are limited. CCA shows relative resistance to both chemotherapy and radiotherapy. Thus chemical treatment is still the primary option [40]. The first-line chemotherapy is based on gemcitabine and cisplatin, and the second-line treatment is the FOLFOX regiment [41,42,43,44]. It is also worth noting that less applied but promising options have arisen, e.g., liver transplantation has become an alternative in iCCA. Multicentre studies have shown that liver transplantation preceded by chemotherapy results ina 5-year disease-free survival (DFS) rate of 65% [45,46]. Immunotherapy alone did not show efficiency in CCA. To date, clinical trials have evaluated the benefits of combining chemotherapy and immunotherapy [47,48]. Concurrently, the discovery of the CCA genome enabled targeting tumour-specific mutations as a palliative therapy option for advanced stages of the disease [49,50]. FGFR inhibitors are mainly considered for iCCA treatment as disorders of this pathway appear most frequently in this type of CCA [51]. Many clinical trials showed the benefits of targeting, especially of FGFR2 [52]. Pemigatinib, a potent, selective inhibitor of FGFR1–3, was approved by FDA based on its beneficial effect in patients with advanced or metastatic CCA that had been previously treated [8]. The results of numerous clinical trials in favour of molecular-targeted therapy have intensified research into potential FGFR inhibitors used in CCA. However, it is necessary to explore the topic with a focus on the effectiveness of such an approach, the potential adverse effects, and resistance to therapy [53].

## 3. Genetic Aberrations in Cholangiocarcinoma

**Figure 2:**
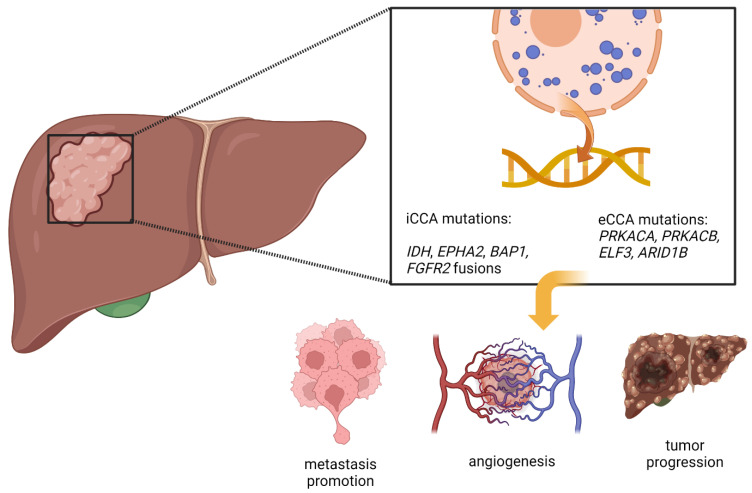
Mutations in CCA, both in iCCA and eCCA, and processes to which they lead.

In this review, the focus remains mainly on the FGFR pathway and genes related to its activation. However, CCA is a tumour with a high mutation burden. Thus, some of these mutations have become desirable therapy targets. Nakamura et al. showed that 40% of CCA cases harboured genetic alterations; approximately 39 non-synonymous mutations per tumour in iCCA and 35 in eCCA [54]. Lowery et al. tested 195 samples of CCA, highlighting that the most frequently altered genes in CCA were *IDH1* (30%), *ARID1A* (23%), *BAP1* (20%), *TP53* (20%), and *FGFR2* gene fusions (14%) [55]. Less common yet significant mutations are in the *RAS*, *PTEN*, and *APC* genes, found in 1–6% of tumours [55,56,57]. The landscape of genetics and epigenetics varies across iCCA and eCCA, as many studies have documented [54,55,58]. While iCCA is mainly characterised by *IDH*, *EPHA2*, and *BAP1* mutations and *FGFR2* fusions, extrahepatic tumours express mainly *PRKACA* and *PRKACB* fusions along with mutations in *ELF3* and *ARID1B* [59,60] (Figure 2). Interestingly, harboured mutations vary not only depending on their localisation but also their etiopathogenesis. Thus, *TP53* and *ARID1A* mutations are more common in fluke-related CCA [57,61]. At the same time, specific mutations correlate with crucial carcinogenesis pathways, e.g., *PTEN* with the RAS–RAF–MAPK pathway or *APC* with the WNT pathway. Moreover, recent years have shown that the origin of CCA carcinogenesis can also be found in epigenetic modulation. Growing evidence supports the thesis that dysregulated methylation may play a crucial role in the impaired differentiation of bile duct epithelium [62]. Azpitarte et al. demonstrated that the *SOX17* promoter is downregulated in CCA compared to healthy tissue [62]. This downregulation activated WNT-dependent proliferation and led to decreased survival among patients after tumour resection [62]. Other studies highlight that histone modifications and aberrant expression of non-coding RNAs can also disturb the balance and cell homeostasis as a result of malignant transformation [63,64,65]. Taking into account all of the alterations mentioned above, Jusakul et al. divided CCA into four clinically significant clusters: fluke-positive CCAs (clusters 1/2) characterised by *ERBB2* amplifications and *TP53* mutations; fluke-negative CCAs (clusters 3/4) with PD-1/PD-L2 expression; epigenetic mutations; and *FGFR* gene rearrangements [58]. Clusters underline the basis of possible use for targeted molecular therapies in CCAs. To sum up, a broad spectrum of mutations in CCA creates multiple possibilities for novel therapies. Therefore, in current clinical trials, a pivotal role is played by *IDH* mutations and *FGFR2* fusions [66], which have found a lasting place in treatment guidelines [67].

## 4. FGF/FGFR Interplay in Cholangiocarcinoma

**Figure 3:**
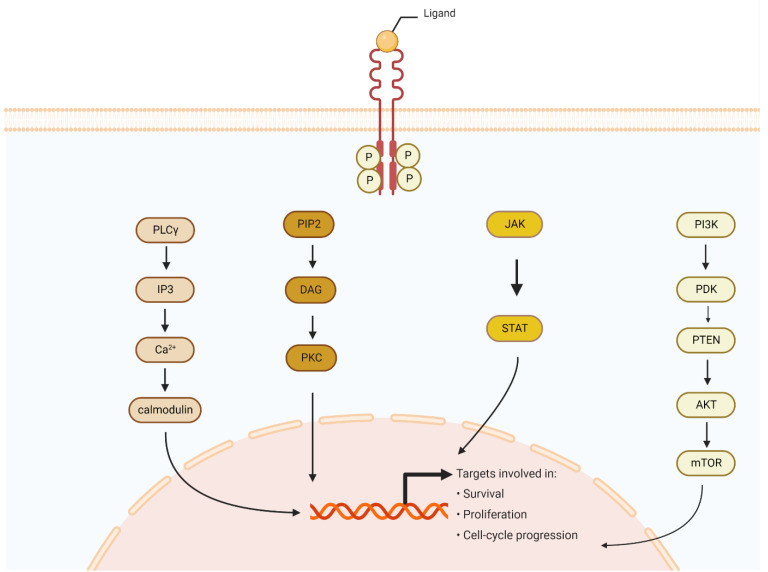
The FGF signalling pathway. The ligand binds to an FGFR monomer, which leads to dimerization and intracellular phosphorylation. This provides the means to start signalling pathways for FGFRs. Activated FGFRs open the way for PI3K, AKT, mTOR or the RAS/RAF/MEK/MAPK cascade. Activated FGFRs also phosphorylate JAK kinases, which lead to STAT activation. FGFRs can also recruit and phosphorylate PLCγ, thereby initiating signalling through the DAG/PKC or IP3-Ca2+ pathway. All of those pathways have a crucial role in tumour development. FGFR (fibroblast growth factor receptors); PI3K (phosphoinositide 3-kinase); AKT (protein kinase B); mTOR (mammalian target of rapamycin); JAK (Janus kinase); STAT (signal transducer and activator of transcription); PLCγ (phospholipase C gamma); DAG (dystroglycan); PKC (protein kinase C); IP3 (inositol trisphosphate).

The family of FGFRs consists of four tyrosine kinase receptors, FGFR1-4. They play an essential role in carcinogenesis and a range of physiological signalling pathways. At the early stages of embryonic development, FGFRs are primarily engaged in fundamental cellular interactions and functions [68,69,70]. It is also known that fundamental metabolic functions are controlled by this pathway, such as the regulation of bile acid, fatty acid, glucose, and mineral metabolism [71,72]. Furthermore, FGF23 and FGFR regulation is crucial for bone development and homeostasis by controlling systemic phosphate homeostasis and vitamin D metabolism [73]. Interestingly, angiogenesis can be stimulated by FGFR both in physiological and neoplastic processes. Activation of FGFR1 or FGFR2 has been demonstrated to affect vascular endothelial proliferation positively [74]. At the same time, four FGF receptors need eighteen FGF ligands that activate them for proper signalling [75]. FGFRs are folded with three extracellular-binding ligand domains, a transmembrane domain, and an intracellular tyrosine kinase domain [75]. Depending on the cell characteristics, extracellular domain D3 can alternatively be spliced and formatted as the epithelial (“b” form) or mesenchymal (“c” form) [76]. Interestingly, novel studies showed the presence of FGFR5/FGFRL1 with unknown functions and different morphology [77,78]. Activation of the receptor results in downstream signalling covering pivotal cellular pathways. Binding the proper ligand to FGFR monomers results in dimerization and intracellular phosphorylation with conformational changes. Activated FGFRs phosphorylate FRS2, which opens the way for PI3K, AKT, mTOR, or the RAS/RAF/MEK/MAPK cascade. Activated FGFRs also phosphorylate JAK kinases, which lead to STAT activation. FGFRs can also recruit and phosphorylate PLCγ, thereby initiating signalling through the DAG/PKC or IP3-Ca2+ pathway. Those pathways have a crucial role in tumour development [79,80,81]. The final effects of these pathways are distinct for the cell and include mitogenesis in the MAPK pathway, cell survival in the PI3K pathway, and mobility in the PKC pathway [82] (Figure 3).

The described pathways are present in CCA as well. Dysregulation at any point may result in the initiation of carcinogenesis, regulate tumour cell proliferation, and activate antiapoptotic pathways or chronic inflammation in bile ducts [83,84,85]. Multiple studies covering different types of cancers showed that FGFRs have a strict correlation with tumour growth and tumour cell proliferation [86,87]. For instance, Sungeun et al. proved that FGFR2 promotes breast cancer tumorigenicity by maintaining tumour-initiating cells [87]. Moreover, the FGFR1 signalling pathway may be crucial in tumour cell invasion [88]. As an essential step in cancer development, angiogenesis can also be affected by FGFR pathway dysregulation [89]. Wang et al. showed that blocking FGFR1 could completely prevent the growth of tumours by blocking angiogenesis [90]. To sum up, every pivotal step in cancer development can be affected crucially by gene amplification or gain-of-function coding mutations, leading to dysregulation of the FGF/FGFR signalling pathway.

As previously mentioned, around 14% of CCA are defined by *FGFR2* fusions, which may lead to the development of this cancer. Moreover, in many cases, mutations of *FGFR1* and *FGFR3* were found, as well as overexpression of *FGFR4* [91]. *FGFR4* overexpression and *FGFR2* alteration are the most important genetic alterations in CCA. Xu et al. showed that this overexpression might lead to the proliferation and invasion of CCA cells in vitro after FGF19 stimulation [92]. The most characteristic translocations of *FGFR2* genes result in constant activation of the pathway. Multiple studies documented that the most prevalent partners in these fusions are *BICC1*, *PPHLN1*, *TACC3*, and *MGEA5* [93]. Ross et al. indicated that the *FGFR2*-*BICC1* fusion results in the abbreviation of the 3′UTR of *FGFR2* and probably an upregulation of the FGFR2 protein [94]. The *FGFR2*-*TACC3* mutation was found in CCA by Borad et al. At the same time, inhibition by pazopanib showed efficacy in inhibiting tumour growth [95]. Sia et al. reported a novel *FGFR2*-*PPHLN1* fusion in CCA, based on the chromosomal translocation t(10;12)(q26;q12), which has both transforming and oncogenic activity [96]. The characteristic *FGFR2* mutations in CCA can not only be targeted in therapy but also predictb patient prognosis. Pu et al. showed that low-level amplification of *FGFR2* implies specific tumour features such as mass-forming, improved overall survival (OS), and lower stage [97]. The novel study documented that the exact type of fusion and its protein products may directly influence therapy results. Protein products of *FGFR2* fusions can be classified into three subtypes: classical fusions that retain the tyrosine kinase (TK) and the Immunoglobulin (Ig)-like domains; sub-classical fusions that retain only the TK domain; and non-classical fusions that lack both [98]. Interestingly, the kinase-deficient fusion lost its sensitivity to FGFR-specific inhibitors [98]. Moreover, Yoo et al. tested 46 iCCAs and found that FGFR4-related genes were significantly associated with improved DFS in iCCA [99].

## 5. Targeted Therapies

Considering the quantity of possible FGFRs alterations in CCA and their pivotal role, not only in molecular targeted therapy but subsequently in prognosis and possible resistance to the treatment, the primary focus should also be on further clinical trials using multiple available inhibitors. Currently, intensive research is conducted not only in the field of monotherapy, but also in multimodal approaches. To date, the most intensively studied molecules are ponatinib, debio 1347, derazantinib, erdafitinib, infigratinib, futibatinib, and pemigatinib. Pemigatinib, infigratinib, and futibatinib are already approved by the FDA as a second-line therapy for advanced CCA [100]. Furthermore, based on the results of clinical trials, molecules can be divided into: non-selective inhibitors (such as lenvatinib, pazopanib, regorafenib, and dovitinib); and novel selective inhibitors (infigratinib, derazantinib, erdafitinib, pemigatinib, futibatinib, and debio 1347) [101,102]. Selective FGFR inhibitors allowed the reduction of side effects resulting from the inhibition of other kinases. Thus their means of action are similar, and rely on reversable bonding with a highly conserved P-loop cysteine residue in an ATP pocket [6,103,104]. The main highlights and information covering specific inhibitors are described below (Table 1).

### 5.1. Ponatinib

Ponatinib is a third-generation kinase inhibitor, primarily applied in chronic myeloid leukaemia (CML) in every phase of the disease and Philadelphia chromosome-positive acute lymphoblastic leukaemia (ALL) [105]. Phase 2 PACE trial proved that this small molecule showed efficiency in inhibiting native and mutant BCR-ABL1, including BCR-ABL1^T315I^; at the same time, the estimated 5-year survival was 73% [106]. The capability of inhibiting numerous tyrosine kinases became the basis of trials with ponatinib in CCA. The first pilot study with ponatinib was completed in early 2022. Ahn et al. included patients with advanced or refractory CCA with FGFR alterations; the primary endpoint was overall response rate (ORR), and secondary endpoints were OS and progression-free survival (PFS) with Health-Related Quality of Life (HRQoL) assessment [107]. The research established partial response in 1 out of 12 patients. Median PFS was 2.4 months, and median OS was 15.7 months. Toxicities were mild and tolerable, with the most common being rash, fatigue, and lymphopenia [107]. Considering this novel approach, more trials with bigger patient groups are needed to establish ponatinib’s place in CCA treatment.

### 5.2. Debio 1347

Debio 1347 is a highly selective, oral FGFR1-3 inhibitor. Primarily, it was tested in various solid tumours with FGFR aberrations, including CCA. The study aimed to establish the tolerated dose (NCT01948297), which was reported to be 80 mg daily with acceptable side effects and encouraging results [108]. In the phase II trial, a daily dose of 80 mg was administered to the patients, including five with CCA. Debio 1347 was well tolerated; furthermore, in the group with FGFR2 fusions, two patients had stable disease (SD), and two patients achieved partial response (PR). The patient with an FGFR1 fusion did not respond to treatment and showed progressive disease (PD) [109]. In 2019, the FUZE clinical trial started recruitment for evaluation of Debio 1347 for patients with advanced, progressive solid tumours (NCT03834220), however low antitumor activity resulted in termination of this study in 2022 [110].

### 5.3. Derazantanib

Derazantanib (ARQ087) is another oral FGFR inhibitor tested in patients with CCA. This molecule is a pan-FGFR inhibitor simultaneously able to inhibit several kinases, such as RET, VEGFR1, DDR, and KIT. The Phase I study (NCT01752920) estimated a dose of 300 mg daily as recommended for the phase II study [111]. Subsequently, an open-label phase I/II trial (NCT01752920) conducted in 29 patients with iCCAs harbouring FGFR2 fusion reported a disease control rate (DCR) and ORR of 82.8% and 20.7%, respectively [112]. Following the results, an open-label, single-arm, phase II FIDES-01 (NCT03230318) trial of derazantinib 300 mg is now ongoing. The trial enrolled previously treated iCCA patients with various FGFR alterations. The primary endpoint to assess the antitumor activity of derazantanib is the proportion of patients with PFS at three months [113].

### 5.4. Erdafitinib

Erdafitinib (Balversa™/JNJ-42756493) is an oral small molecule with activity against all four FGFRs and other related kinases (e.g., VEGFR) to a lesser extent [114]. During the phase IIa study conducted in China, Korea, and Taiwan (NCT02699606), adults with advanced CCA harbouring FGFR alterations who had failed at least one prior systemic treatment received erdafitinib 8 mg daily. Strict observations and dose escalation depended on phosphate levels. Among 17 enrolled patients, 15 had a significant response to treatment, 7 achieved PR, and 5 had SD. The ORR was 47%, and the DCR was 80% [115].

### 5.5. Infigratinib

Infigratinib (BGJ398) is an oral ATP-competitive FGFR1–3-selective inhibitor [116]. The first evaluation stage was a dose-escalation and dose-expansion study with patients with advanced malignancies harbouring FGFR genetic aberrations (NCT01004224). According to the results of the phase II study, the recommended dose for the FGFR inhibitor was 125 mg once daily (three weeks on, one week off schedule) [117]. The final discussed trial conducted by Javle et al. showed that an ORR of 23.1%, with median duration of response of 5.0 months and a median PFS of 7.3 months, was achieved among 108 cases of pre-treated CCA patients with *FGFR2* fusion or rearrangement [118]. The most common treatment-emergent adverse events (TEAEs) were hyperphosphatemia, eye disorders, stomatitis, and fatigue. Based on the results of clinical trials on 28 May 2021, the FDA granted accelerated approval to infigratinib for adults with previously treated, unresectable, locally advanced or metastatic CCA with *FGFR2* fusion or another rearrangement [7]. Currently, further trials are being conducted, including the PROOF-301 phase III study of infigratinib versus chemotherapy which has a chance to establish the new first-line, chemotherapy-free, targeted therapy option for these patients (NCT03773302) [119].

### 5.6. Futibatinib

Futibatinib (TAS-120) is the only oral FGFR1-4 selective inhibitor with a unique mechanism of action, binding covalently and irreversibly to FGFR [120]. Sootome et al. evaluated the anti-cancer activity of futibatinib, whose oral administration led to significant dose-dependent tumour reduction in various FGFR-driven human tumour xenograft models [121]. Furthermore, the frequency of drug-resistant clones was lower with futibatinib than with a reversible ATP-competitive FGFR inhibitor, and futibatinib inhibited several drug-resistant FGFR2 mutants [121]. These results may indicate the potential use of futibatinib in cases of resistance to other FGFRs inhibitors. The FOENIX-101 first-in-human, phase I dose-escalation trial (NCT02052778) evaluated the safety of futibatinib in advanced solid tumours [122]. A daily dose of 20 mg was established as the recommended phase II dose, with PR reported in five patients and SD in 41 [123]. FOENIX-CCA2, an open-label, multicentre phase II registrational trial in patients with iCCA harbouring FGFR2 gene fusions or other rearrangements (NCT02052778), was conducted based on FOENIX-1 results. The initial outcomes from the FOENIX-CCA2 study were reported from 103 patients who had progressed on previous standard therapies or for whom standard therapy was not tolerated [124]. The ORR was 37.3%, and the DCR was 82.1% [124]. Furthermore, based on the results of FOENIX trials, futibatinib was approved by FDA this year for the treatment of locally advanced or metastatic cholangiocarcinoma whose tumours harbor an *FGFR2* rearrangement or fusion [125]. There is a FOENIX-CCA3 trial planned for futibatinib versus chemotherapy with gemcitabine and cisplatin as the first-line treatment in patients with *FGFR2* alterations (NCT04093362) [126].

### 5.7. Pemigatinib

Pemigatinib is the first molecule used in targeted therapy for CCA and was first approved for treatment in 2020 [127]. Pemigatinib is an oral selective inhibitor of FGFR1–3, with weaker activity against FGFR4 [128]. In pre-clinical models, Liu et al. showed that, even with the use of small oral doses, pemigatinib suppressed the growth of xenografted tumour models with FGFR1, 2, or 3 alterations [129]. This therapeutic agent was proven efficient both in monotherapy and in combination with cisplatin [129]. The phase I/II FIGHT-101 trial evaluated pemigatinib in patients with previously treated solid tumours with or without FGFR aberrations (NCT02393248) [130]. The estimated daily dose was 13.5 mg, and no dose-limiting toxicities were observed. Pemigatinib showed both efficacy and tolerability in monotherapy and in combination with other drugs [130]. The FIGHT-101 trial became the cornerstone for the FIGHT-202 trial, which enrolled patients with CCA harbouring FGFR2 gene fusions or rearrangements, other FGFR aberrations, or without FGFR aberrations (NCT02924376) [8]. Outlining the main findings, Abou et al. showed that 35% of patients with *FGFR2* fusions or rearrangements had an objective response, including three cases with complete response. Moreover, median PFS and median OS of 6.9 months and 21.1 months were achieved, respectively. The main adverse events associated with this therapy were hypophosphatemia (12%), arthralgia (6%), stomatitis (5%), hyponatremia (5%), abdominal pain (5%), and fatigue (5%). Nevertheless, spectacular results in the cohort with *FGFR2* fusions grew to be the foundation for the future use and approval of this compound [8]. At the same time, the phase III trial (FIGHT-302) with pemigatinib versus chemotherapy as the first-line treatment in CCA is ongoing (NCT03656536) [131].

**Table 1:** Current status of FGFRi in clinical development for CCA.

	The Current Stage of Development	Inhibitor Generation/Potency	Efficacy Results	Adverse Events and Disadvantages of the Therapy	NCT/Reference
Ponatinib	The first study was conducted, based on the results of 12 patients with CCA.	Third-generation TKI; FGFR1-4; VEGFR2; RET; c-KIT; BCR-ABL1	mPFS 2.4 months; mOS 15.7 months	Rash, fatigue, lymphopenia	[107]
Debio 1347	Two main clinical trials with mixed results; phase II study showed great results in patients with CCA, however, the FUZE study has been terminated due to low antitumor activity.	Third generation TKI; highly selective for FGFR1-3	mPFS 18.3 weeks	fatigue, hyperphosphatemia, anaemia, alopecia, nausea, vomiting, constipation, and palmar-plantar erythrodysesthesia syndrome	NCT01948297 [108,109]
Derazantanib	Trial with hopeful results followed by ongoing FIDES-01 trial with tumours harbouring *FGFR2* alterations.	FGFR1-3, RET, VEGFR1, DDR, KIT	mPFS 5.7 months	hyperphosphatemia, dry mouth and nausea, asthenia, fatigue, dysgeusia, vomiting, dry eye, conjunctivitis, blurred vision, photophobia	NCT01752920 NCT03230318 [111,112,113]
Erdafitinib	Trial for patients harbouring *FGFR2* mutations in the Asian population.	First-generation TKI inhibitor; FGFR1-4 and to lesser extent VEGFR	mPFS 2.35 months	Hyperphosphatemia, stomatitis, dry mouth, elevated AST, elevated ALT	NCT02699606 [115]
Infigratinib	Approved by FDA for unresectable, locally advanced, or metastatic CCA with *FGFR2* fusion or another rearrangement. Ongoing phase III trial versus chemotherapy in patients with CCA.	FGFR1-3 selective inhibitor	mPFS 7.3 months	hyperphosphatemia, eye disorders, stomatitis, and fatigue	NCT01004224 NCT03773302 [117,118,119]
Futibatinib	Approved by FDA for locally advanced or metastatic CCA harbouring an *FGFR2* rearrangement or fusion. Phase III FOENIX-CCA3 trial recruiting.	FGFR1-4 selective inhibitor	mPFS 9 months	Hyperphosphatemia, diarrhoea, dry mouth	NCT02052778 NCT04093362 [122,124,125,126]
Pemigatinib	Approved by FDA for previously treated, unresectable, advanced/ metastatic CCA with *FGFR2* alterations. Phase III trial (FIGHT-302) versus chemotherapy as first-line treatment in CCA is ongoing.	FGFR1-3 and weaker activity against FGFR4	mOS 21.1 months mPFS 6.9 months	Hyperphosphatemia, alopecia, diarrhoea, fatigue, dysgeusia	NCT02393248 NCT02924376 NCT03656536 [8,128,129,130]

## 6. Key Questions and How to Address Them

In the last decade, FGFR inhibitors have become an integral part of CCA treatment, eventually permanently entering the guidelines. However, two main issues should be addressed after analysing data from both pre-clinical and clinical studies. Firstly, most patients with *FGFR2* mutations failed to achieve an overall response. Moreover, the median duration of response was only 5–6 months [132]. That may indicate both primary and acquired resistance, as seen in different types of cancer [9]. Secondly, the vast majority of FGFR inhibitors showed numerous side effects in clinical trials, which often lead to treatment discontinuation. To incorporate FGFR inhibitors even more efficiently on a treatment basis, those key questions about resistance and disadvantages must be answered.

### 6.1. Primary and Acquired Resistance Mechanisms

Primary resistance is often expressed in specific fusions with other co-occurring tumour-suppressing genes. As stated by Silverman et al., there is a tendency in CCA towards a shorter progression-free survival amongst patients with *FGFR2* fusions with *BAP1*, *CDKN2A*/*B*, *PBRM1*, and *TP53* [133]. Furthermore, Person et al. showed that primary resistance might depend on *FGFR* amplification in various cancers [134]. Significant response to treatment was seen only in high-level *FGFR*-amplified cancers, with copy-number level dictating the response to FGFR inhibition in vitro, in vivo, and in the clinic [134].

Acquired resistance was mainly observed in clinical trials, which resulted from incomplete or no response in some cases. In 2017, Goyal et al. for the first time reported for the first time the genetic mechanisms of clinically-acquired resistance to FGFR inhibition in patients with *FGFR2* fusion-positive ICC [135]. The study, conducted with serial analysis of cell-free circulating tumour DNA (cfDNA) from three patients, showed that acquired resistance to infigratinib is correlated with point mutations in the FGFR2 kinase domain during progression [135]. The most common mutation found in every sample was the p.V565F gate-keeper mutation, and two patients developed polyclonal secondary mutations in the FGFR2 kinase domain. Furthermore, this research documented that cfDNA analysis can distinguish more evident mutations than a single tumour biopsy, concluding that heterogeneity of the tumour possibly plays a role in the resistance to FGFR inhibitors [135]. Moreover, other mutations in *FGFR* may lead to resistance. For instance, tumour cells harbouring activating V561M mutation in the FGFR1 kinase domain showed resistance to both specific inhibitors AZD4547 and infigratinib; and non-specific inhibitors, such as ponatinib, TKI258, and lucitanib (E3810) [136]. Interestingly, other pathways correlated with FGFR can be involved in the secondary resistance mechanism. Thus, Cowell et al. showed that mutational inactivation of *PTEN* resulted in increased PI3K/AKT activity and resensitization to FGFR inhibitors [136]. Moreover, Datta et al. documented that AKT activation mediates resistance to infigratinib, and that adding an AKT inhibitor or small interfering RNA (siRNA) can restore sensitivity to infigratinib in resistant cell lines [137]. The above-mentioned mechanisms indicate the urgent need for investigation into the use of combined therapies in CCA to overcome this resistance. However, another solution may lie in an inhibitor with a unique mechanism of action; futibatinib. Futibatinib showed significant activity in CCA with *FGFR2* gene fusions, and efficacy in patients with progression on prior FGFR inhibitors. Goyal et al. showed that futibatinib led to clinical benefits in patients primarily treated with infigratinib or Debio 1347, overcoming several *FGFR2* mutations in iCCA models [138]. Futibatinib retained activity against several mutations by altering the conformational dynamics of FGFR2. In the analysis of the most common mutations in cell lines, futibatinib was active against all except the *FGFR2* p.V565F gatekeeper mutation [138]. Furthermore, a clinical trial conducted in 2018 (NCT02052778) in 45 patients previously treated with chemotherapy or prior FGFR inhibitors showed definitive clinical activity of futibatinib against resistance to primary therapy [122]. Tran et al. proved that in 28 patients with *FGFR2* gene fusions, 20 (71%) experienced tumour shrinkage, and 7 had confirmed partial responses [122].

### 6.2. Crucial Disadvantages of FGFR-Targeted Therapy

Molecular targeted therapy was intended to bring less systematic toxic effects compared to chemotherapy. However, the FGFR signalling pathway is also involved in many cellular physiological processes, hence this approach’s numerous side effects. Even though FGFR inhibitors are well-tolerated, these drugs are associated with disadvantages that are distinct from other small-molecule tyrosine kinase inhibitors and other drugs used in this indication. As reported in clinical trials, these toxicities can result in dose reductions, interruptions, and even drug discontinuation. Hyperphosphatemia is most commonly associated with this therapy, resulting from the influence of FGFR1 and FGF23 on the organism’s phosphate metabolism [81,139]. Clinical trials showed that up to 60% of patients are affected by this complication; however, rarely did these patients experience grade ≥ 3 side effects [102]. There are numerous management options for patients who develop hyperphosphatemia during the treatment, including dietary changes with a low-phosphate diet and phosphate-binding agents [53]. This approach allows limiting-dose reduction or discontinuation of the treatment. Ophthalmological toxicity includes retinal pigment epithelial detachment (RPED) and central serous retinopathy (CSR) as the most severe, though rare (5%), complication; but more commonly, dry eye occurs in clinical trials (19–21%) [8,66,124]. Unfortunately, if symptoms are significant, they may be the reason for discontinuing the therapy. Moreover, dermatological toxicities, including hand-foot syndrome, hair loss, nail-bed infections, onycholysis, dry skin, and xerostomia, may occur [8,66]. Management of these adverse events is based on symptomatic treatment, using glucocorticosteroids, antibiotics, topical urea, nail avulsion, and improving the patient’s quality of life [140]. Indeed, significant results are achieved with FGFR inhibitorsbut the side-effect profile may limit their utility. Therefore, particular attention should be focused on preventing and effectively managing FGFR-inhibitor-induced adverse events.

## 7. Conclusions

The occurrence of CCA is mainly associated with the mutations that lead to the upregulation of the FGF/FGFR signalling pathway. Thus, researchers continuously strive to develop such inhibitors and targeted therapies that would specifically inhibit the carcinogenic effects of this disturbed pathway. Three molecular-specific drugs are already approved, while other therapies are still undergoing investigation. Considering current interventions, the use of FGFR inhibitors seems to be beneficial. However, a potential side effect of therapy should be addressed before accepting this therapy into the canon of practice. Another issue constitutes the molecular characterization of a patient’s CCA to introduce the most effective therapeutic approach. Furthermore, some tumours might develop drug resistance during therapy, significantly decreasing the overall clinical outcome with the necessity of implementing other treatment strategies. For this reason, there is a need for other drugs to be investigated in clinical trials, especially novel inhibitors with different mechanisms of action, such as futibatinib, whose irreversible mechanism of action has proven to be effective in clinical trials. Combined therapies, in the first place, are more effective managing the CCA. Moreover, they allow for the minimization of potential side effects. The breakthrough would allow the development of therapies that could inhibit the major carcinogenic pathway leading to break-off CCA growth and progression.

In conclusion, FGFR inhibitors have taken a permanent place in the treatment of many cancers, especially CCA, allowing for more favourable treatment outcomes. However, despite the huge therapeutic success, the range of side effects and relapses have become their main limitation. The solution seems to be to look further for new molecular targets and new drug combinations if we want to minimalize the devastating impact of CCA.

## Data Availability

Not applicable.

## Abbreviations

CCA	cholangiocarcinoma
iCCA	intrahepatic cholangiocarcinoma
pCCA	perihilar cholangiocarcinoma
dCCA	distal cholangiocarcinoma
eCCA	extrahepatic cholangiocarcinoma
FGFR	fibroblast growth factor receptor
FGF	fibroblast growth factor
TK	tyrosine kinase
TKI	tyrosine kinase inhibitor
FDA	Food and Drug Administration
IDH1	Isocitrate dehydrogenase 1
ARID1A/ARID1B	AT-Rich Interaction Domain 1A/1B
BAP1 BRCA1	Associated Protein 1
TP53	tumour protein p53
RAS	rat sarcoma viral proto-oncogene
PTEN	Phosphatase And Tensin Homolog
APC	Regulator Of WNT Signalling Pathway
EPHA2	Epithelial Cell Receptor Protein Tyrosine Kinase A2
PRKACA/PRKACB	Protein Kinase CAMP-Activated Catalytic Subunit Alpha/Beta
ELF3 E74	Like ETS Transcription Factor 3
PD-1/PD-L1	Programmed cell death protein 1/Programmed death-ligand 1
ERBB2	Erb-B2 Receptor Tyrosine Kinase 2
PI3K	phosphoinositide 3-kinase
AKT	kinases protein kinase B family
mTOR	Mammalian target of rapamycin
PLCγ	phospholipase C gamma
DAG	dystroglycan
PKC	protein kinase C
RAF	rapidly accelerated fibrosarcoma kinase
MEK	Mitogen-activated protein kinase kinase
MAPK	Mitogen activated protein kinase
JAK	kinase Janus kinase
STAT	signal transducer and activator of transcription
IP3	inositol trisphosphate
BICC1	Protein Bicaudal C Homolog 1
PPHLN1	Periphilin 1
TACC3	Transforming Acidic Coiled-Coil Containing Protein 3
MGEA5	meningioma expressed antigen 5
RET	Ret Proto-Oncogene
VEGFR1	Vascular endothelial growth factor receptor 1
DDR DNA	damage response and repair gene
CDKN2A/B	cyclin-dependent kinase inhibitor 2A/B
PBRM1	Polybromo 1 gene
cfDNA	cell-free circulating tumour DNA
siRNA	small interfering RNA
OS	overall survival
DFS	disease-free survival
ORR	overall response rate
PFS	progression-free survival
SD	stable disease
PR	partial response
PD	progressed disease
DCR	disease control rate
TEAE	treatment-emergent adverse events
AE	adverse events
